# Alemtuzumab improves contrast sensitivity in patients with relapsing–remitting multiple sclerosis

**DOI:** 10.1177/1352458513475722

**Published:** 2013-09

**Authors:** Jennifer Graves, Steven L Galetta, Jeffrey Palmer, David H Margolin, Marco Rizzo, John Bilbruck, Laura J Balcer

**Affiliations:** 1 University of Pennsylvania, Philadelphia, PA, USA; 2 University of California San Francisco (UCSF), CA, USA; 3 Genzyme Corporation, Cambridge, MA, USA; 4 UBC-Envision Group, Horsham, UK

**Keywords:** Multiple sclerosis, relapsing–remitting multiple sclerosis, outcome measurements, disease modifying therapy, alemtuzumab, interferon beta, contrast sensitivity, visual function

## Abstract

**Background:**

Alemtuzumab is a monoclonal antibody directed against CD52 that depletes T and B lymphocytes.

**Objective:**

To evaluate the treatment effect of alemtuzumab on low-contrast vision in relapsing–remitting multiple sclerosis (RRMS) patients.

**Methods:**

This was a pre-defined exploratory analysis within a randomized, rater-blinded trial (CAMMS223) that was run at 49 academic medical centers in the US and in Europe. Patients with untreated, early, RRMS (McDonald, *n* = 334) were randomized 1:1:1 to subcutaneous interferon beta-1a (IFNB-1a), or alemtuzumab 12 mg or 24 mg. Visual contrast sensitivity was measured for each eye at baseline and quarterly, with Pelli-Robson charts.

**Results:**

The eyes of patients in the pooled alemtuzumab group (versus IFNB-1a) had a greater than 2-fold higher rate of both 3-month and 6-month sustained visual improvement, of at least 0.3 log units (2 triplets, 6 letters) (At 3 months the hazard ratio (HR) = 2.26; CI = 1.19 to 4.31; *P* = 0.013; and at 6 months the HR = 2.44; CI =1.16 to 5.15; *P* = 0.019), and they had a lower risk of 3- and 6-month sustained worsening of at least 0.15 log units (1 triplet, 3 letters) (At 3 months the HR = 0.58; CI = 0.38 to 0.89; *P* = 0.012; and at 6 months HR = 0.55; CI=0.35 to 0.87; *P* = 0.010). Over the 36-month study period, the eyes of patients in the pooled alemtuzumab group improved in mean contrast sensitivity to a greater extent than those in the IFNB-1a group (0.080 log units versus 0.038 log units; *P* = 0.0102).

**Conclusions:**

Alemtuzumab was associated with a greater chance of improved contrast sensitivity in patients with RRMS and may delay the worsening of visual function. Contrast sensitivity testing was sensitive to treatment effects, even within an active comparator study design. These results support the validity of low-contrast vision testing as a clinical outcome in MS trials.

## Introduction

Visual dysfunction occurs in 80% of patients with multiple sclerosis (MS) at some point in the disease and 20% present with acute optic neuritis.^1–3^ In addition, MS patients frequently show subclinical changes in visual function with no apparent visual symptoms.^4–6^ Such impairments can remain undetected, due to the lack of sensitivity of some visual function tests, particularly those based solely on high- contrast acuity.^7^ Tests measuring low-contrast vision (with shades of grey on a white background) are more sensitive clinical measures of visual dysfunction in MS^8–10^ and can detect abnormalities even in MS patients with otherwise apparently good visual acuity.^11,12^ Pelli-Robson charts measure contrast sensitivity, the lowest contrast level at which patients can perceive letters of a single large size (~20/680 Snellen equivalent). This method was used in the Optic Neuritis Treatment Trial, and was a practical and sensitive indicator of visual dysfunction in optic neuritis.^13^

Abnormalities in low contrast vision, in addition to correlating with measures of visual disability, have been shown to be predictive for changes in overall MS disability and functionality and to be significantly associated with magnetic resonance imaging (MRI) abnormalities, such as brain atrophy.^14^ Low-contrast letter acuity charts measure the smallest size at which patients can see letters at different levels of contrast. Scores on this test correlate well with structural biomarkers of axonal and neuronal loss, including retinal nerve fibre layer thickness and macular volume, as measured by optical coherence tomography.^15,16^ Therefore, measures of low contrast vision are potentially powerful and practical tools to measure the effectiveness of MS therapies. Studies also demonstrate that low-contrast letter acuity testing may have potential utility as an additional component to disability scales such as the Multiple Sclerosis Functional Composite.^17^

Alemtuzumab is a monoclonal antibody that selectively targets CD52, to deplete circulating T and B lymphocytes, the critical mediators of MS inflammatory processes, while having minimal impact on the other immune cells.^18^ A distinctive pattern of T and B cell repopulation begins within weeks, leading to a rebalancing of the immune system. Although the exact mechanism of action of alemtuzumab in MS is unknown, these pharmacodynamic changes may help explain its disease-modifying effects on MS.^18^ In the Phase II CAMMS223 trial, alemtuzumab reduced the risk of relapse and of sustained accumulation of disability (SAD), compared to subcutaneous high-dose interferon beta-1a (IFNB-1a) in treatment-naïve relapsing–remitting multiple sclerosis (RRMS) patients with active disease.^19^ There was a mean improvement in MS-related disability reported.^18,20^ Alemtuzumab is also associated with infusion-associated reactions, infection and autoimmunity (primarily thyroid disease and immune thrombocytopenia).

Measurement of low contrast vision using Pelli-Robson charts was an exploratory efficacy outcome of CAMMS223, with the resultant data presented here.

## Methods

CAMMS223 was a 36-month Phase II randomized, rater-blinded trial, investigating two dose levels of alemtuzumab versus subcutaneous IFNB-1a, the details of which were previously published.^19^ CAMMS223 is registered at ClinicalTrials.gov, as number NCT00050778. All procedures were approved by the local institutional ethics review boards of the participating sites. Patients provided written informed consent.

### Patients

Patients (*n* = 334) with early active RRMS and no prior disease-modifying therapy were enrolled at 49 centers in Europe and North America, between December 2002 and July 2004. An independent safety-monitoring committee oversaw the running of the trial. The study data collected by investigators were held and analyzed by Genzyme Corporation, working with the authors.

Eligible patients had a diagnosis of RRMS based on the McDonald criteria,^21^ with an onset of symptoms no more than 36 months preceding screening, at least two clinical episodes (MS relapses) during the previous 2 years, a score of 3 or less on the Expanded Disability Status Scale (EDSS)^22^ and at least one gadolinium-enhancing lesion, as seen on at least one of up to four monthly cranial MRI scans. Patients were excluded if they received previous disease-modifying treatment, had a history of clinically-significant autoimmunity other than MS, or had positive serum anti-thyrotropin-receptor antibodies.

### Treatments

Eligible patients were randomly assigned, in a 1:1:1 ratio, to receive an alemtuzumab dosage of 12mg or 24mg, or subcutaneous IFNB-1a. Alemtuzumab was given by intravenous infusion on five consecutive days during the first month, and on three consecutive days at month 12. Some patients had a further treatment for three consecutive days, at month 24. IFNB-1a was self-administered subcutaneously three times weekly, at a dose of 44 μg, following dose escalation.

### Assessments

Pelli-Robson contrast sensitivity charts contain large letters (20/680 Snellen equivalent at 1m), which decrease in contrast, but not in size. Each group of three letters has the same contrast, with successive groups decreasing in contrast by a factor of 1/√2, from a very high level down to a contrast that is below the threshold of recognition for normal observers. The subject reads the letters on the chart, starting with those of the highest contrast. A subject’s threshold for contrast sensitivity is taken to be the lowest contrast for which at least two letters in a triplet group are correctly reported.^9^

A key is provided with the Pelli-Robson chart, where the triplets identified correctly are translated into a log sensitivity value and each triplet is worth 0.15 log. For example, the triplet with 1/100 contrast (1%) has a log contrast sensitivity of 2.00.^16^ “Normal” levels for Pelli-Robson contrast sensitivity were previously described as 1.80 log units for younger patients and 1.65 log units for older patients.^23^

Visual assessment using contrast sensitivity testing was a pre-defined exploratory endpoint in the CAMMS223 study. Pelli-Robson testing was carried out for each eye individually, at baseline and thereafter at quarterly intervals for 3 years, by a clinical professional who was blinded to the MS treatment assignment. Pelli-Robson testing was not performed at some Eastern European study sites where the native language used the Cyrillic alphabet, for which no Pelli-Robson translation exists. For an eye, a clinically significant sustained improvement in the contrast score is defined as a ≥ 0.30 log unit increase in contrast sensitivity (equivalent to two triplets, or six letters) that is sustained for at least 3 months.^23^ Worsening of contrast sensitivity in an eye is defined as a decrease of 0.30 log units, but given that there were too few of these events to permit interpretation, decreases of 0.15 log units (three letters) sustained for 3 months were also analyzed. Also, additional analyses were carried out to assess 6-month sustained changes.

### Statistical analysis

The study’s patient cohort was composed of all randomized patients, who were diagnosed with MS at entry into the study. Comparisons of the time to sustained improvement or sustained worsening in each eye were analyzed using a Cox proportional-hazards regression model and Kaplan-Meier estimation,^24^ adjusted for within-patient inter-eye correlations, and was defined as the time in days from randomization to the first date when a change in contrast sensitivity occurred that was maintained at the next follow-up assessment.

The mean change in contrast sensitivity from the baseline time point was assessed, using a mixed model for repeated measures^25^ with study visit and treatment group interaction terms included as covariates, and with adjustment for within-patient inter-eye correlations.

Relationships between the change in Pelli-Robson score and the change in EDSS or the change in EDSS Visual Functional System Score (FSS) were assessed, using Spearman rank correlation coefficients.

As per the overall study analyses, the 12 mg and 24 mg alemtuzumab dose groups were both pooled for analysis and also analyzed separately. All statistical models were adjusted for baseline EDSS, baseline contrast sensitivity for the right and left eyes, age at baseline, country and history of optic neuritis (yes/no) as covariates. No adjustments were made for multiple hypothesis testing. All reported *p*-values were 2-sided.

## Results

A total of 273 randomized patients (90 receiving IFNB-1a, 91 receiving alemtuzumab 12 mg, 92 receiving alemtuzumab 24 mg) were included in the contrast sensitivity analyses. Details of the study cohorts, and the overall efficacy and safety of alemtuzumab, have been published previously.^19,26^ Baseline demographic and clinical characteristics of the patients included in this analysis, including baseline contrast sensitivity scores in Table 1, were balanced between the treatment groups and were similar to the full study cohorts (as reported elsewhere^19^). A history of optic neuritis at study entry was noted for 36 patients (40.0%) in the IFNB-1a group, 30 (33.0%) in the alemtuzumab 12 mg group and 44 (47.8%) in the alemtuzumab 24 mg group.

**Table 1.** Baseline demographics and clinical disease characteristics of patients included in the contrast sensitivity analyses.

	**IFNB-1a**	**Alemtuzumab 12 mg**	**Alemtuzumab 24 mg**	**Alemtuzumab pooled**	P-value^*^
	N=90	N=91	N=92	N=183	
**Age, mean years (SD)**	33.6 (9.2)	32.4 (8.0)	32.9 (8.6)	32.7 (8.3)	0.6352
**Gender, % female**	70.0	72.5	67.4	69.9	0.7515
**Race, % Caucasian**	87.8	89.0	87.0	88.0	0.9462
**Baseline EDSS, mean (SD)**	1.85 (0.83)	1.90 (0.75)	1.96 (0.75)	1.93 (0.76)	0.6551
**Time since first episode, median years (min, max)**	1.35 (0.2, 6.3)	1.20 (0.1, 3.5)	1.20 (0.3, 3.2)	1.20 (0.1, 3.5)	0.3952
**Relapses in 2 years prior to baseline, n (%)**					
**0**	0	2 (2.2)	0	2 (1.1)	
**1**	7 (7.8)	5 (5.5)	10 (10.9)	15 (8.2)	0.1333
**2**	58 (64.4)	47 (51.6)	46 (50.0)	93 (50.8)	
**≥3**	25 (27.8)	37 (40.7)	36 (39.1)	73 (39.9)	
**Baseline log contrast sensitivity, mean (SD)**					
**Left eye**	1.60 (0.24)	1.58 (0.26)	1.59 (0.20)	1.58 (0.23)	0.8672
**Right eye**	1.60 (0.20)	1.61 (0.24)	1.61 (0.18)	1.61 (0.21)	0.8901
**History of optic neuritis^†^**, **n (%)**	36 (40.0)	30 (33.0)	44 (47.8)	74 (40.4)	0.1231

* P-values are from tests of imbalance among the IFNB-1a, alemtuzumab 12 mg, and alemtuzumab 24 mg treatment groups. Categorical variables are analyzed using Fisher’s exact test, and continuous variables are analyzed using a one-way Analysis of Variance.

† A patient was considered to have a history of optic neuritis if “reduced visual acuity” had been reported in either the left or right eye during any of their prior clinical relapse episodes.

In Table 2 we see that the eyes of patients in the pooled alemtuzumab group showed a greater improvement from baseline to month 36 (0.080 log units; *P* < 0.0001), when compared to those in the IFNB-1a group (0.038 log units; *P* = 0.0316), representing a net difference of 0.042 log units (*P* = 0.0102). The change in mean contrast sensitivity for each of both alemtuzumab dose groups versus IFNB-1a were also statistically significant (12 mg: difference = 0.041 log units, *P* = 0.0285; 24 mg: difference = 0.043 log units, *P* = 0.0189).

**Table 2.** Mean change in contrast sensitivity from baseline to Month 36.

	IFNB-1a (n=180)	Alemtuzumab 12 mg (n=182)	Alemtuzumab 24 mg (n=183)	Pooled alemtuzumab (n=365)
Adjusted analyses*				
**Change from Baseline to Month 36, log units (95% CI)**	0.038 (0.003, 0.072)	0.079 (0.047, 0.110)	0.081 (0.050, 0.111)	0.080 (0.054, 0.106)
**P-value**	0.0316	<0.0001	<0.0001	<0.0001
**Difference^†^, log units**		0.041 (0.004, 0.078)	0.043 (0.007, 0.079)	0.042 (0.010, 0.074)
**P-value**		0.0285	0.0189	0.0102

* Estimates, 95% confidence intervals, and p-values are from a mixed model for repeated measures with covariate adjustment for baseline contrast sensitivity, baseline EDSS score, age, country, and history of optic neuritis.

† Estimated difference between each alemtuzumab treatment group and IFNB-1a from the mixed model for repeated measures.

**Figure 1.**
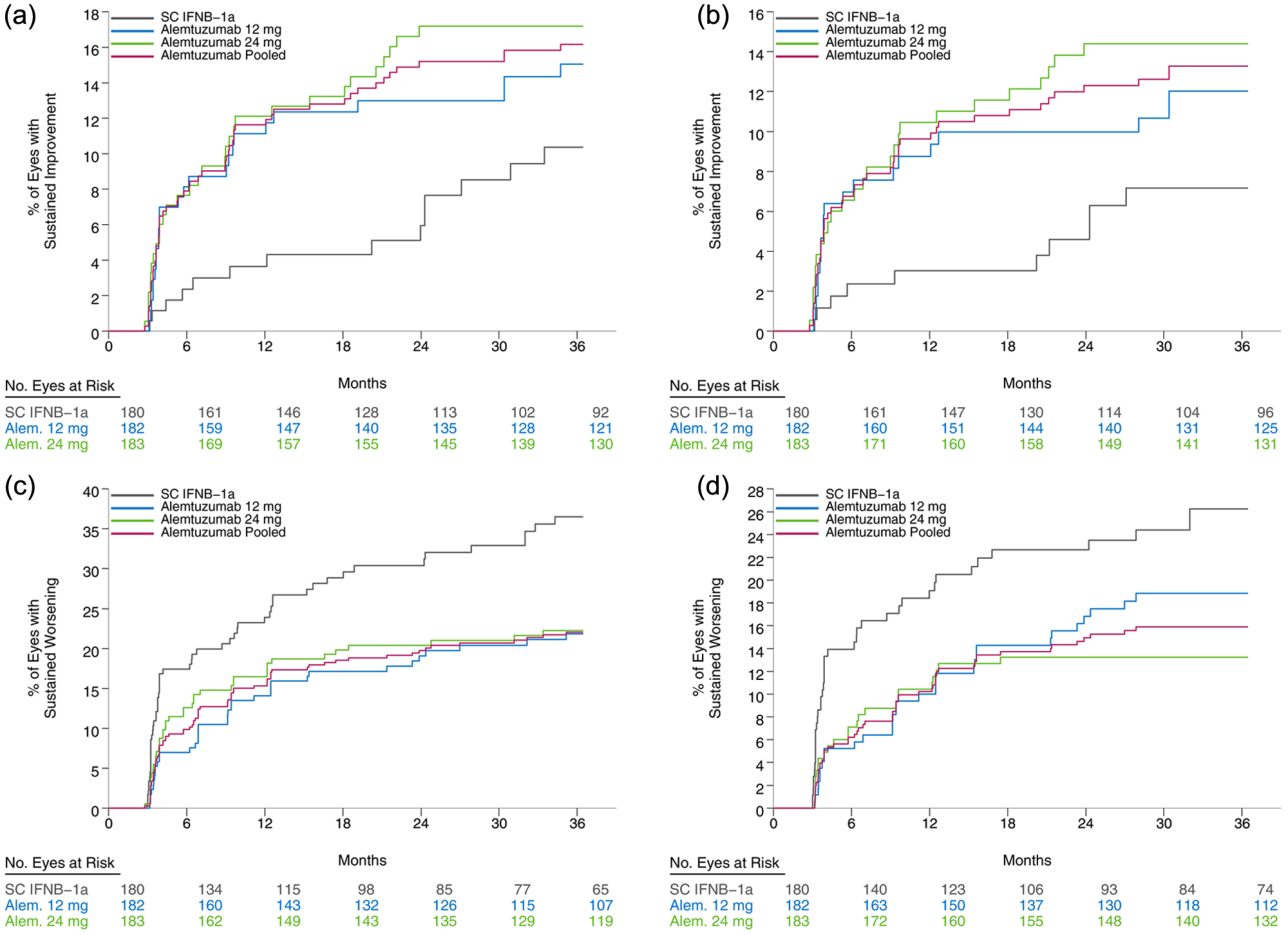
Kaplan-Meier estimates of time to improvement or worsening in visual contrast sensitivity by MS treatment group: (a) sustained improvement (3 months, 0.30 log units), (b) sustained improvement (6 months, 0.30 log units), (c) sustained worsening (3 months, 0.15 log units) and (d) sustained worsening (6 months, 0.15 log units). Alem.: Alemtuzumab; IFNB: interferon beta; MS: multiple sclerosis; SC: subcutaneous dose

The differences in the time to sustained improvement of at least 0.30 log units in visual contrast sensitivity for 3 months (see Figure 1(a) and Table 3) and for 6 months (see Figure 1(b) and Table 3) were appreciable between the alemtuzumab and IFNB-1a treatment groups. Data for the eyes of pooled alemtuzumab patients showed that they were more than twice as likely to have 3- and 6-month sustained improvements in vision, as compared to those receiving IFNB-1a (Table 3). Analysis of the different doses of alemtuzumab showed that patients in the 24 mg dosage group had higher rates of both 3- and 6-month sustained improvements than those in the IFNB-1a group (3 months, *P* = 0.0064; 6 months, *P* = 0.0055). While the vision of patients receiving alemtuzumab 12 mg was also more likely to improve than those receiving IFNB-1a, we found that the difference between these groups did not reach nominal statistical significance levels.

**Table 3.** Time to sustained improvement of 0.30 log units in eyes.

	IFNB-1a (n=180)	Alemtuzumab 12 mg (n=182)	Alemtuzumab 24 mg (n=183)	Pooled alemtuzumab (n=365)
	**3 Months Sustained Improvement**			
**Eyes with event, n**	14	25	31	56
**Kaplan-Meier estimate of event, % (95% CI)**	10.4 (5.8, 18.1)	14.9 (9.8, 22.2)	17.2 (11.8, 24.7)	16.1 (12.2, 21.1)
Hazard ratio* (95% CI)		1.937 (0.947, 3.963)	2.667 (1.317, 5.400)	2.304 (1.194, 4.446)
P-value*		0.070	0.0064	0.0128
	**6 Months Sustained Improvement**			
**Eyes with event, n**	10	20	26	46
**Kaplan-Meier estimate of event, % (95% CI)**	7.2 (3.7, 13.8)	11.9 (7.2, 19.3)	14.4 (9.6, 21.3)	13.2 (9.6, 18.0)
Hazard ratio* (95% CI)		1.983 (0.805, 4.885)	3.184 (1.407, 7.205)	2.587 (1.167, 5.734)
P-value*		0.1366	0.0055	0.0193

* P-values from a Cox proportional-hazards regression model with baseline left and right eye contrast sensitivity scores, country, baseline age, baseline EDSS and history of optic neuritis included as covariates in the model.

Sustained worsening of at least 0.30 log units was also assessed; however, deterioration of this magnitude occurred for very few eyes in each treatment group. An estimated 7% of eyes in both the pooled alemtuzumab and IFNB-1a treatment groups had a sustained worsening of 0.30 log units for a period of 3 months. Sustained worsening of at least 0.15 log units was less likely to occur in the eyes of patients in the pooled alemtuzumab treatment groups, as compared with those in the IFNB-1a group, when using both the 3-month (*P* = 0.0087) (Figure 1(c) and Table 4) and 6-month criteria (*P* = 0.0084) (Figure 1(d) and Table 4). The eyes of patients receiving either dose of alemtuzumab were significantly less likely to have 3-month sustained deterioration, as compared to those receiving IFNB-1a (12 mg, *P* = 0.020; 24 mg, *P* = 0.038). The patients in the alemtuzumab 24 mg dosage group also had a lower likelihood of worsened vision than those in the IFNB-1a group, using the 6-month criterion.

**Table 4.** Time to sustained worsening of 0.15 log units in eyes.

	IFNB-1a (n=180)	Alemtuzumab 12 mg (n=182)	Alemtuzumab 24 mg (n=183)	Pooled alemtuzumab (n=365)
	**3 Months Sustained Worsening**			
**Eyes with event, n**	56	36	40	76
**Kaplan-Meier estimate of event, % (95% CI)**	36.5 (27.7, 47.0)	21.6 (15.0, 30.4)	22.3 (15.8, 30.8)	21.9 (17.1, 27.9)
Hazard ratio* (95% CI)		0.557 (0.341, 0.911)	0.588 (0.356, 0.970)	0.573 (0.378, 0.869)
P value*		0.0197	0.0377	0.0087
	**6 Months Sustained Worsening**			
**Eyes with event, n**	41	31	24	55
**Kaplan-Meier estimate of event, % (95% CI)**	26.2 (18.8, 35.9)	18.6 (12.7, 26.8)	13.2 (18.2, 21.0)	15.8 (11.7, 21.2)
Hazard ratio* (95% CI)		0.665 (0.403, 1.097)	0.443 (0.241, 0.814)	0.546 (0.348, 0.857)
P value*		0.1104	0.0087	0.0084

* P-values from a Cox proportional-hazards regression model with baseline left and right eye contrast sensitivity scores, country, baseline age, baseline EDSS and history of optic neuritis included as covariates in the model.

In a sensitivity analysis that was restricted to the patients who completed the study, the estimated proportion of patients with sustained improvement or worsening in visual contrast sensitivity was similar to that observed in the overall analysis (data not shown). Results from statistical models that were unadjusted for covariates were consistent with the covariate-adjusted analyses reported here.

Changes in visual contrast sensitivity did show some correlation with the subjects’ performance on the Visual FSS component of the EDSS. The changes from baseline to month 12 in contrast sensitivity, for all eyes, were correlated with the changes in Visual FSS during the same time period (correlation coefficient (*ρ*) = −0.12; *p* = 0.0059). There was also a correlation for the change from baseline to month 24 (*ρ* = −0.21; *p* < 0.0001), but no association was found between change in contrast sensitivity and change in Visual FSS from baseline to month 36 (*ρ* = −0.076; *p* = 0.13). These correlations were of modest magnitude, even when statistically significant, and no correlation was observed between the changes in Pelli-Robson scores and changes from baseline in the overall EDSS score (data not shown).

## Discussion

This study demonstrates that alemtuzumab may be associated with better visual outcomes, compared with IFNB-1a, and that the Pelli-Robson measurement of low contrast vision is sensitive to treatment effects. Interestingly, the patients treated with IFNB-1a also demonstrated improvements in low contrast vision, but without a placebo arm to the study, the significance of this observation is unclear.

It is important to consider the benefits of the observed treatment effect. Contrast sensitivity is a critical aspect of visual function, as it measures how well a person can see under low contrast conditions, such as driving at night or reading in low ambient light. Visual impairment of this nature can significantly reduce quality of life, making people more dependent on others for transport and causing difficulty with carrying out daily tasks.^27,28^ The inability to detect, for instance, the edge of a step, can be a contributory factor in accidents.^29,30^ In patients with MS, reductions in quality of vision can produce impairments in vision- specific health-related quality of life measures that are similar in magnitude to those caused by having glaucoma or cataracts.^31^

Our study demonstrated that Pelli-Robson charts may detect treatment effects in a controlled trial. Contrast sensitivity tests are found to be abnormal, even in MS patients with Snellen acuities of 20/20 or better.^10,12,17,32,33^ Visual impairment may occur independent of changes in EDSS.^8^ Contrast letter acuity (Sloan charts) and contrast sensitivity (Pelli-Robson system) are the methods that best distinguish visual dysfunction in patients with MS, compared with disease-free controls.^32^ Low-contrast letter acuity scores were shown to be predictive for changes in MS disability and functionality.^33^ In this study, no association was found to exist between Pelli-Robson scores and the overall EDSS changes from baseline. This observation confirmed that visual changes may occur independently of EDSS changes and that contrast sensitivity changes may demonstrate neurological dysfunction not captured by the visual function system score of the EDSS.

Limitations in the current analysis are acknowledged. Although this endpoint was pre-specified in the protocol, aspects of the analyses described were post hoc in nature. Pelli-Robson assessments were not performed for every patient in the CAMMS223 study. Among those patients who had a baseline contrast sensitivity measurement, 60.0% of IFNB-1a and 86.8% of alemtuzumab patients participated in the full study period and had a month 36 evaluation. This difference in study drop-out rates between the treatment groups could potentially bias the observed treatment effects; however, a sensitivity analysis restricted to patients who completed the study showed that the estimated proportion of patients with sustained improvement or worsening was similar to the results from the overall analysis, which suggested that the results reported here are likely consistent with the population as a whole, in spite of the differential drop-out rates. Binocular measurements, which could better reflect visual function in a natural setting, were not made in this study. Pre-baseline practice tests of contrast sensitivity were not part of the protocol. While learning effects are a possible limitation, such effects would tend to increase the scores for all patients, including those in the interferon-treated group. This would be expected to limit the sensitivity of the within-group comparison, but not invalidate any observed between-group differences.

In summary, the results of this Phase 2 study suggested that alemtuzumab therapy improves the contrast sensitivity in RRMS patients better than SC IFNB-1a does. These findings support previous analyses in demonstrating the capacity of low-contrast vision tests to measure treatment effects in MS patients.^34^ Given their validity in measuring visual dysfunction, coupled with their being non-invasive, inexpensive and time-efficient, and their correlation with MRI and optical coherence tomography (OCT) structural markers, the tests of low-contrast vision are practical outcome measures to consider in a MS trial setting.

## References

[1] Frohman EM Frohman TC Zee DS The neuro-ophthalmology of multiple sclerosis. Lancet Neurol 2005; 4: 111–121 15664543 10.1016/S1474-4422(05)00992-0

[2] McDonald WI Barnes D The ocular manifestations of multiple sclerosis. 1. Abnormalities of the afferent visual system. J Neurol Neurosurg Psych 1992; 55: 747–752 10.1136/jnnp.55.9.747 PMC1015095 1402963

[3] Newman NJ Multiple sclerosis and related demyelinating diseases. In: Miller NR Newman NJ (eds) Walsh and Hoyt’s clinical neuro-opthalmology. Nth ed Baltimore: Williams & Wilkins, 1998: 5th edition; pages 5539–5676

[4] Sisto D Trojano M Vetrugno M Subclinical visual involvement in multiple sclerosis: A study by MRI, VEPs, frequency-doubling perimetry, standard perimetry, and contrast sensitivity. Invest Ophthalmol Vis Sci 2005; 46: 1264–1268 15790888 10.1167/iovs.03-1213

[5] Lycke J Tollesson PO Frisén L Asymptomatic visual loss in multiple sclerosis. J Neurol 2001; 248: 1079–1086 12013586 10.1007/s004150170029

[6] Engell T Trojaborg W Raun NE Subclinical optic neuropathy in multiple sclerosis. A neuro-ophthalmological investigation by means of visually evoked response, Farnworth-Munsell 100 Hue test and Ishihara test and their diagnostic value. Acta Ophthalmol (Copenh) 1987; 65: 735–740 3434241 10.1111/j.1755-3768.1987.tb07073.x

[7] Balcer LJ Frohman EM Evaluating loss of visual function in multiple sclerosis as measured by low-contrast letter acuity. Neurology 2010; 74: S16–23 20421569 10.1212/WNL.0b013e3181dbb664

[8] Balcer LJ Baier ML Pelak VS New low-contrast vision charts: Reliability and test characteristics in patients with multiple sclerosis. Mult Scler 2000; 6: 163–171 10871827 10.1177/135245850000600305

[9] Pelli DG Robson JG Wilkins AJ The design of a new letter chart for measuring contrast sensitivity. Clin Vision Sci 1988; 2: 187–199

[10] Regan D Neima A Low-contrast letter charts as a test of visual function. Ophthalmol 1983; 90: 1192–1200 10.1016/s0161-6420(83)34407-9 6657195

[11] Kupersmith MJ Nelson JI Seiple WH The 20/20 eye in multiple sclerosis. Neurology 1983; 33: 1015–1020 6683795 10.1212/wnl.33.8.1015

[12] Nordmann JP Saraux H Roullet E Contrast sensitivity in multiple sclerosis: A study in 35 patients with and without optic neuritis. Ophthalmologica 1987; 195: 199–204 3431817 10.1159/000309813

[13] Trobe JD Beck RW Moke PS Contrast sensitivity and other vision tests in the optic neuritis treatment trial. Am J Ophthalmol 1996; 121: 547–553 8610798 10.1016/s0002-9394(14)75429-7

[14] Wu GF Schwartz ED Lei T Relation of vision to global and regional brain MRI in multiple sclerosis. Neurology 2007; 69: 2128–2135 17881718 10.1212/01.wnl.0000278387.15090.5a

[15] Fisher JB Jacobs DA Markowitz CE Relation of visual function to retinal nerve fiber layer thickness in multiple sclerosis. Ophthalmol 2006; 113: 324–332 10.1016/j.ophtha.2005.10.040 16406539

[16] Osborne B Jacobs D Markowitz C Relation of macular volume to retinal nerve fiber layer thickness and visual function in multiple sclerosis. Neurology 2006; 66: A14

[17] Balcer LJ Baier ML Cohen JA Contrast letter acuity as a visual component for the Multiple Sclerosis Functional Composite. Neurology 2003; 61: 1367–1373 14638957 10.1212/01.wnl.0000094315.19931.90

[18] Coles A Cox A Le Page E The window of therapeutic opportunity in multiple sclerosis: Evidence from monoclonal antibody therapy. J Neurol 2006; 253: 98–108 16044212 10.1007/s00415-005-0934-5

[19] CAMMS223 Trial Investigators, Coles AJ Compston DA Alemtuzumab vs. interferon beta-1a in early multiple sclerosis. N Engl J Med 2008; 359: 1786–1801 18946064 10.1056/NEJMoa0802670

[20] Fox EJ Sullivan HC Gazda SK A single-arm, open-label study of alemtuzumab in treatment refractory patients with multiple sclerosis. Eur J Neurol 2012; 19: 307–311 21899662 10.1111/j.1468-1331.2011.03507.x

[21] McDonald WI Compston A Edan G Recommended diagnostic criteria for multiple sclerosis: Guidelines from the international panel on the diagnosis of multiple sclerosis. Ann Neurol 2001; 50: 121–127 11456302 10.1002/ana.1032

[22] Kurtzke JF Rating neurologic impairment in multiple sclerosis: An Expanded Disability Status Scale (EDSS). Neurology 1983; 33: 1444–1452 6685237 10.1212/wnl.33.11.1444

[23] Elliott DB Sanderson K Conkey A The reliability of the Pelli-Robson contrast sensitivity chart. Ophthalmic Physiol Opt 1990; 10: 21–24 2330208

[24] Ying Z Wei LJ The Kaplan-Meier estimate for dependent failure time observations. J Multivar Anal 1994; 50: 17–29

[25] Mallinckrodt CH Clark WS David SR Accounting for dropout bias using mixed-effects models. J Biopharm Stat 2001; 11: 9–21 11459446 10.1081/BIP-100104194

[26] Coles AJ Fox E Vladic A Alemtuzumab versus interferon beta-1a in early relapsing–remitting multiple sclerosis: Post-hoc and subset analyses of clinical efficacy outcomes. Lancet Neurol 2011; 10: 338–348 21397567 10.1016/S1474-4422(11)70020-5

[27] McGwin G Chapman V Owsley C Visual risk factors for driving difficulty among older drivers. Accid Anal Prev 2000; 32: 735–744 10994600 10.1016/s0001-4575(99)00123-2

[28] Owsley C Stalvey BT Wells J Visual risk factors for crash involvement in older drivers with cataract. Arch Ophthalmol 2001; 119: 881–887 11405840 10.1001/archopht.119.6.881

[29] Lord S Dayhew J Visual risk factors for falls in older people. J Am Geriatr Soc 2001; 49: 508–515 11380741 10.1046/j.1532-5415.2001.49107.x

[30] Lord SR Menz HB Visual contributions to postural stability in older adults. Gerontology 2000; 46: 306–310 11044784 10.1159/000022182

[31] Ma SL Shea JA Galetta SL Self-reported visual dysfunction in multiple sclerosis: New data from the VFQ-25 and development of an MS-specific vision questionnaire. Am J Ophthalmol 2002; 133: 686–692 11992867 10.1016/s0002-9394(02)01337-5

[32] Weinstock-Guttman B Baier M Stockton R Pattern reversal visual evoked potentials as a measure of visual pathway pathology in multiple sclerosis. Mult Scler 2003; 9: 529–534 14582782 10.1191/1352458503ms935rr

[33] Baier ML Cutter GR Rudick RA Low-contrast letter acuity testing captures visual dysfunction in patients with multiple sclerosis. Neurology 2005; 64: 992–995 15781814 10.1212/01.WNL.0000154521.40686.63

[34] Balcer LJ Galetta SL Calabresi PA Natalizumab reduces visual loss in patients with relapsing multiple sclerosis. Neurology 2007; 68: 1299–1304 17438220 10.1212/01.wnl.0000259521.14704.a8

